# Mol­ecular structure, DFT studies and UV–Vis absorption of two new linear fused ring chalcones: (*E*)-1-(anthracen-9-yl)-3-(2-meth­oxy­phen­yl)prop-2-en-1-one and (*E*)-1-(anthracen-9-yl)-3-(3-fluoro-4-meth­oxy­phen­yl)prop-2-en-1-one

**DOI:** 10.1107/S205698901800974X

**Published:** 2018-07-13

**Authors:** Dian Alwani Zainuri, Ibrahim Abdul Razak, Suhana Arshad

**Affiliations:** a X-ray Crystallography Unit, School of Physics, Universiti Sains Malaysia, 11800 USM, Penang, Malaysia

**Keywords:** chalcone, anthracene, crystal structure, DFT, UV–Vis

## Abstract

The title compounds, C_24_H_18_O_2_ and C_24_H_17_FO_2_, were synthesized using the Claisen–Schmidt condensation method and characterized by UV–Vis spectroscopy. Weak inter­molecular C—H⋯O, C—H⋯π and π–π hydrogen-bonding inter­actions help to stabilize the crystal structures of both compounds. The geometrical parameters obtained from the mol­ecular structure were optimized using density functional theory (DFT) calculations at the B3LYP/6–311++G(d,p) level, showing a good correlation with the experimental results. The small HOMO–LUMO energy gaps of 3.11 and 3.07 eV enhances the non-linear responses of these mol­ecular systems.

## Abstract 2

The title compounds were synthesized using the Claisen–Schmidt condensation method and characterized by UV–Vis spectroscopy. The geometrical parameters optimized using density functional theory (DFT) calculations show a good correlation with the experimental results. The small HOMO–LUMO energy gaps of 3.11 and 3.07 eV enhances the non-linear responses of these mol­ecular systems.

## Chemical context   

Conjugated organic systems contain delocalized π electrons, which often show excellent NLO properties as they can easily be polarized. There are three features essential for high non-linear activity in an organic compound which are: a strong electron donor, a highly polarizable π-conjugated bridged moiety and a strong π-electron acceptor. Chalcones generally satisfy these criteria given their π-conjugated bridged structures that can be functionalized with a wide range of substitutions. Recently, we found that the presence of an anthracene fused-ring system positioned at the terminal ring of these derivative compounds is useful in getting good quality single crystals with an easily synthesizable method. The structure of anthracene is benzene-like, having three six-membered rings fused together in a planar-like arrangement. These polyaromatic hydro­carbons containing π-conjugated materials show unique properties in terms of conductivity that have led to significant advancements in the field of organic electronics (Li *et al.*, 2016 ▸). In this work, we report the synthesis and combined experimental and theoretical studies of two new anthracene chalcones C_24_H_18_O_2_ (I) and C_24_H_17_FO_2_ (II), containing methoxyphenyl (I) and fluoromethoxyphenyl (II) groups as substituents. Additionally, the UV–Vis absorption and HOMO–LUMO analysis are also reported herein.

## Structural commentary   

**Figure 1:**
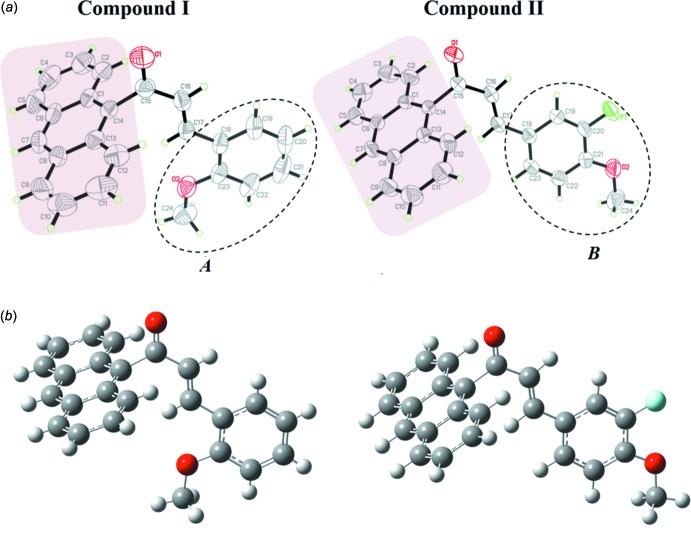
(*a*) The mol­ecular structure for compounds (I) and (II) showing the atom-numbering schemes and 50% probability ellipsoids; (*b*) The DFT-optimized structures at the B3LYP 6–311++G(d,p) level for compounds (I) and (II).

The new chalcones C_24_H_18_O_2_ (I) and C_24_H_17_FO_2_ (II) consist of an anthracene fused-ring system and the substituent units 1-meth­oxy-2-methyl­benzene (*A*) and 2-fluoro-1-meth­oxy-4-methyl­benzene (*B*), respectively. These compounds represent *D*–*A* π inter­molecular charge-transfer systems. Displacement ellipsoid plots and DFT optimized structures of the title compounds with their atom-labeling schemes are shown in Fig. 1 ▸. Compounds (I) and (II) crystallize in the monoclinic *P*2_1_ */c* and triclinic *P* 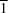 space groups, respectively. Selected B3LYP/6-311++G(d,p) geometry-optimized calculated values (Frisch *et al.*, 2009 ▸) for the bond lengths and angles of both compounds based on geometries in the gaseous state are compared to those of the crystalline structures in the solid state in Table S1 in the supporting information. The theoretical bond lengths and bond angles correlate well with the experimental data and are in normal ranges.
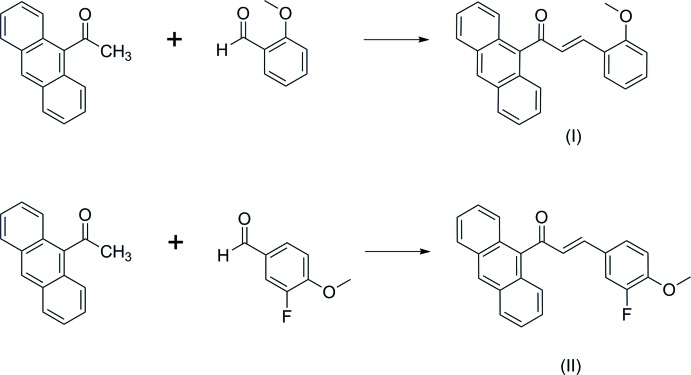


Both mol­ecular structures adopt an *s*-*trans* configuration with respect to the C16=C17 double bond across the ethyl­enic bridge (O1/C15–C17). The anthracene unit in both (I) and (II) is found to be twisted at the C14—C15 bond with the C1—C14—C15—C16 torsion angles being −95.91 (18)° in (I) and −106.3 (2)° in (II). This is probably due to the bulkiness of the strong electron donor. The corresponding DFT-calculated results give values of −95.94° (I) and −91.27°(II), respectively. The experimental and theoretical torsion-angle difference of 15.0° observed in (II) is most likely due to the formation of a weak inter­molecular C12—H12 O2 inter­action involving the anthracene fused-ring system with the terminal meth­oxy substituent unit.

The mean plane of the enone moiety in (I) [O1/C15–C17, maximum deviation of 0.0085 (18) Å at C16] forms dihedral angles of 88.15 (18) and 1.44 (19)° with the mean plane of the anthracene ring system (C1–C14) and the 1-meth­oxy-2-methyl­benzene (*A*) ring, respectively. The DFT geometry-optimization calculations give the same values as the experimental values. In (II) the mean plane of the enone moiety [O1/C15–C17, maximum deviation of 0.0092 (18) Å at C16] forms dihedral angles of 73.65 (18) and 2.40 (19)° with the mean planes of the anthracene ring system (C1–C14) and the 2-fluoro-1-meth­oxy-4-methyl­benzene ring (*B*). The corresponding DFT geometry-optimization calculation gives values of 89.99 and 0.01°, respectively. Additionally, the mean plane of the anthracene ring system (C1–C14) in the two compounds form dihedral angles of 87.52 (8)° (experimental and DFT) and 71.31 (7)° (experimental) and 90.00° (DFT) with the mean planes of *A* and *B*, respectively.

## Supra­molecular features   

**Figure 2:**
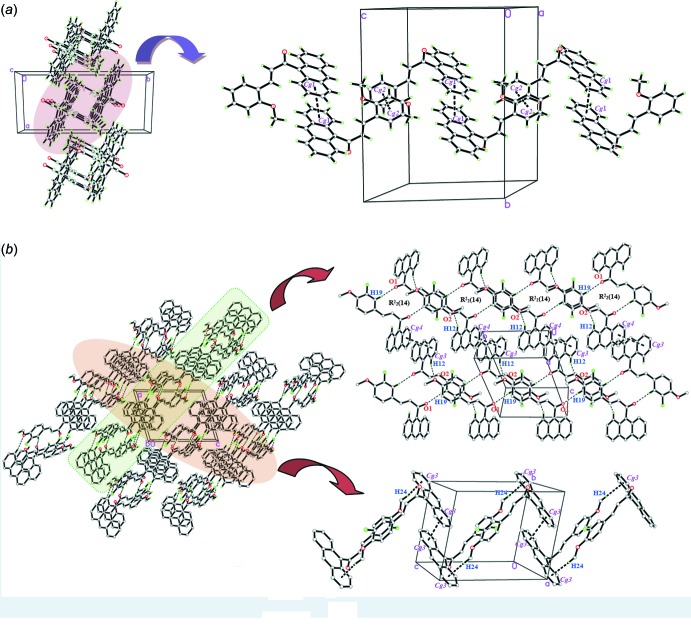
(*a*) Crystal packing for compound (I) viewed along the *a* axis showing weak π–π inter­actions (dashed lines), where *Cg*1 and *Cg*2 are the centroids of the C1–C6 and C18–C22 rings, respectively, and (*b*) Weak C—H⋯ O, C—H⋯π and π–π inter­actions (dashed lines) for compound (II),, forming 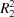
(14) ring graph-set motifs, where *Cg*3 and *Cg*4 are the centroids of the C8–C13 and C1–C6 rings, respectively.

The crystal packing of both compounds is shown in Fig. 2 ▸ and details of the weak inter­molecular hydrogen-bonding inter­actions are given in Table 1 ▸. No classical hydrogen bonds are observed in either structure. The crystal packing of (I) shows only weak π–π inter­actions (Table 2 ▸) with centroid–centroid distances of 3.8804 (12) and 3.6725 (13) Å. The mol­ecules are further linked into infinite zigzag chains along the *c*-axis direction.

In (II), weak C19—H19*A*⋯O1^ii^ and C12—H12*A*⋯O2^i^ hydrogen bonds (Table 1 ▸) connect the mol­ecules into centrosymmetric dimers with 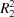
(14) ring motifs. These dimers are further linked into infinite sheets stacked along the *a*-axis direction. Weak C24—H24⋯*Cg*4^iii^ (Table 1 ▸) and π–π inter­actions [centroid–centroid distances = 3.8126 (11) and 3.789 (12) Å; Table 2 ▸] are also observed in the crystal packing and further stabilize the crystal structure. These weak inter­molecular C—H⋯O, C—H⋯π and π–π inter­actions are significant in bridging the mol­ecules into a three-dimensional supra­molecular network.

## UV–Vis absorption analysis   

**Figure 3:**
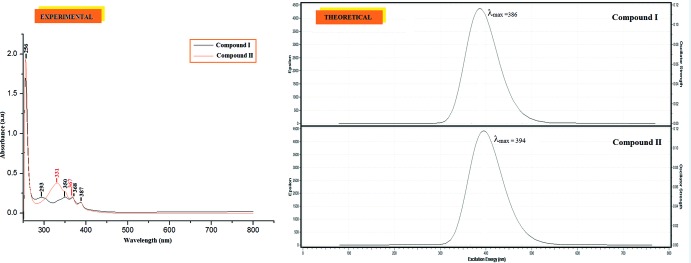
UV–Vis absorption spectra of compounds (I) and (II).

Experimental electronic absorption spectra of (I) and (II) have been measured and compared to the ground state (HOMO) and excited state (LUMO) mol­ecular orbital energies, calculated using time-dependent DFT B3LYP/6-311++G(d,p) theoretical calculations in the gas phase. The experimental absorption peaks (Fig. 3 ▸) of (I) and (II) are found at the same maximum wavelength of 387 nm, whereas the simulated values are observed at 386 nm and 394 nm, respectively. The shift of the theoretical values to higher wavelengths are due to the fact that the calculations are confined to a gaseous environment, whereas the observations are obtained from the solution state (Zainuri *et al.*, 2017 ▸).

**Figure 4:**
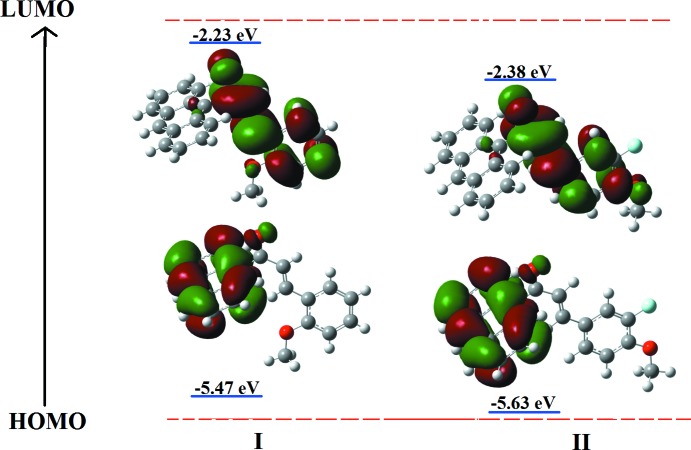
Mol­ecular orbital electron distributions of the HOMO and LUMO energy levels for (I) and (II).

The HOMO and LUMO energies characterize the ability of donating and accepting electrons, whereas the value of the energy gap between the HOMO and LUMO mol­ecular orbitals characterizes the mol­ecular chemical stability. The energy gaps are largely responsible for the chemical and spectroscopic properties of the compounds. In Fig. 4 ▸, the charge densities in the ground state (HOMO) are mainly delocalized over the entire anthrancenyl donor ring, while in the excited state (LUMO), the charge densities are accumulated on the π-conjugated enone bridge and the terminal electron-acceptor group. The HOMO and LUMO energy gaps were computed to be 3.24 eV for (I) and 3.25 eV for (II). Through an extrapolation of the linear trend observed in the optical spectra, the experimental energy band gaps for (I) and (II) become 3.11 eV and 3.07 eV, respectively. These optical band-gap values indicate the suitability of these compounds for opto-electronic applications as for structures of chalcones previously reported by Prabhu *et al.* (2016 ▸).

## Database survey   

A survey of the Cambridge Structural Database (CSD, Version 5.39, last update November 2017; Groom *et al.*, 2016 ▸) revealed several fused-ring substituted chalcones similar to (I) and (II). There are four compounds that have an anthrancene-ketone subtituent on the chalcone, including 9-anthryl styryl ketone and 9,10-anthryl bis­(styryl ketone) reported by Harlow *et al.* (1975 ▸). (2*E*)-1-(Anthracen-9-yl)-3-[4-(propan-2-yl)phenyl]prop-2-en-1-one was reported by Girisha *et al.* (2016 ▸), while (*E*)-1-(anthracen-9-yl)-3-(2-chloro-6-fluoro­phen­yl)prop-2-en-1-one was reported by Abdullah *et al.* (2016 ▸). Zainuri *et al.* (2018*a* ▸) reported a chalcone with two anthrancene substit­uents, *viz.* (*E*)-1,3-bis­(anthracen-9-yl)prop-2-en-1-one. Other related compounds include 1-(anthracen-9-yl)-2-methyl­prop-2-en-1-one (Agrahari *et al.*, 2015 ▸), 9-anthroylacetone (Cicogna *et al.*, 2004 ▸), (*E*)-1-(anthracen-9-yl)-3-(naphthalen-2-yl)prop-2-en-1-one and (*E*)-1-(anthracen-9-yl)-3-(pyren-1-yl)prop-2-en-1-one (Zainuri *et al.*, 2018*b* ▸,*c* ▸).

## Synthesis and crystallization   

A mixture of 9-acetyl­anthracene (0.5 mmol) and 2-meth­oxy­benzaldehyde (0.5 mmol) and 3-fluoro-4-meth­oxy­benzaldehyde (0.5 mmol) for compounds (I) and (II), respectively, was dissolved in methanol (20 ml). A catalytic amount of NaOH (5 ml, 20%) was added to the solutions, dropwise under vigorous stirring. The reaction mixtures were stirred for about 5-6 h at room temperature. After stirring, the contents of the flask were poured into ice-cold water (50 ml). The resultant crude products were filtered, washed successively with distilled water and recrystallized to get the corres­ponding chalcones (see scheme). Single crystals of (I) and (II) suitable for X-ray diffraction were obtained by the slow evaporation technique using acetone.

## Refinement   

Crystal data collection and structure refinement details are summarized in Table 3 ▸. All H atoms were positioned geom­etrically [C—H = 0.93 and 0.96 Å in (I) and (II)] and refined using a riding model with *U*_iso_(H) = 1.2 or 1.5*U*_eq_(C). A rotating group model was applied to the methyl group. In the final refinement of (I), one outlier (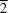 2 15) was omitted.

## Supplementary Material

Crystal structure: contains datablock(s) I, II. DOI: 10.1107/S205698901800974X/jj2199sup1.cif

Structure factors: contains datablock(s) I. DOI: 10.1107/S205698901800974X/jj2199Isup2.hkl

Structure factors: contains datablock(s) II. DOI: 10.1107/S205698901800974X/jj2199IIsup3.hkl

Supporting information file. DOI: 10.1107/S205698901800974X/jj2199Isup4.cml

Supporting information file. DOI: 10.1107/S205698901800974X/jj2199IIsup5.cml

Comparison of selected experimental and DFT-optimized data for compounds (I) and (II). DOI: 10.1107/S205698901800974X/jj2199sup6.pdf

CCDC references: 1827020, 1817223

Additional supporting information:  crystallographic information; 3D view; checkCIF report

## Figures and Tables

**Table 1:** Hydrogen-bond geometry (Å, °) for (II) *Cg*4 is the centroid of the C1–C6 ring.

*D*—H⋯*A*	*D*—H	H⋯*A*	*D*⋯*A*	*D*—H⋯*A*
C12—H12*A*⋯O2^i^	0.93	2.48	3.345 (2)	154
C19—H19*A*⋯O1^ii^	0.93	2.48	3.393 (3)	166
C24—H24*D*⋯*Cg*4^iii^	0.96	2.77	3.391 (3)	123

Symmetry codes: (i) 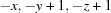; (ii) 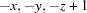; (iii) 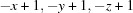.

**Table 2:** Weak π–π inter­actions in compounds (I) and (II) *Cg*1 and *Cg*2 are the centroids of the C1–C6 and C18–C23 rings, respectively, in compound (I). *Cg*3 and *Cg*4 are the centroids of the C8–C13 and C1–C6 rings, respectively, in compound (II).

*I*	*J*	*I*⋯*J*	Symmetry
*Cg*1	*Cg*1	3.8804 (12)	1 − *x*, 2 − *y*, 2 − *z*
*Cg*2	*Cg*2	3.6725 (13)	1 − *x*, 2 − *y*, 1 − *z*
*Cg*3	*Cg*3	3.7891 (12)	1 − *x*, 1 − *y*, 2 − *z*
*Cg*3	*Cg*4	3.8126 (11)	1 − *x*, −*y*, 2 − *z*

**Table 3:** Experimental details

	(I)	(II)
Crystal data		
Chemical formula	C_24_H_18_O_2_	C_24_H_17_FO_2_
*M*_r_	338.38	356.37
Crystal system, space group	Monoclinic, *P*2_1_/*c*	Triclinic, *P* 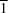
Temperature (K)	294	296
*a*, *b*, *c* (Å)	9.0554 (8), 17.4260 (15), 12.9217 (9)	8.6646 (5), 9.5752 (5), 11.5636 (6)
α, β, γ (°)	90, 119.916 (5), 90	100.593 (2), 105.443 (2), 92.422 (2)
*V* (Å^3^)	1767.3 (3)	904.76 (9)
*Z*	4	2
Radiation type	Mo *K*α	Mo *K*α
μ (mm^−1^)	0.08	0.09
Crystal size (mm)	0.60 × 0.23 × 0.15	0.99 × 0.31 × 0.25

Data collection		
Diffractometer	Bruker SMART APEXII DUO CCD area-detector	Bruker SMART APEXII DUO CCD area-detector
Absorption correction	Multi-scan (*SADABS*; Bruker, 2009 ▸)	Multi-scan (*SADABS*; Bruker, 2009 ▸)
No. of measured, independent and observed [*I* > 2σ(*I*)] reflections	30614, 4249, 2916	34988, 5394, 3307
*R*_int_	0.045	0.045
(sin θ/λ)_max_ (Å^−1^)	0.661	0.711

Refinement		
*R*[*F* ^2^ > 2σ(*F* ^2^)], *wR*(*F* ^2^), *S*	0.054, 0.146, 1.08	0.059, 0.183, 1.02
No. of reflections	4249	5394
No. of parameters	236	245
H-atom treatment	H-atom parameters constrained	H-atom parameters constrained
Δρ_max_, Δρ_min_ (e Å^−3^)	0.17, −0.19	0.27, −0.20

Computer programs: *APEX2* and *SAINT* (Bruker, 2009 ▸), *SHELXL2014* (Sheldrick, 2014), *SHELXTL* (Sheldrick, 2008 ▸) and *PLATON* (Spek, 2009 ▸).

## supplementary crystallographic information

### (I) Crystal data

**Table:**

C_24_H_18_O_2_	*F*(000) = 712
*M**_r_* = 338.38	*D*_x_ = 1.272 Mg m^−^^3^
Monoclinic, *P*2_1_/*c*	Mo *K*α radiation, λ = 0.71073 Å
*a* = 9.0554 (8) Å	Cell parameters from 6535 reflections
*b* = 17.4260 (15) Å	θ = 2.6–28.0°
*c* = 12.9217 (9) Å	µ = 0.08 mm^−^^1^
β = 119.916 (5)°	*T* = 294 K
*V* = 1767.3 (3) Å^3^	Needle, yellow
*Z* = 4	0.60 × 0.23 × 0.15 mm

### (I) Data collection

**Table:**

Bruker SMART APEXII DUO CCD area-detector diffractometer	2916 reflections with *I* > 2σ(*I*)
Radiation source: fine-focus sealed tube	*R*_int_ = 0.045
φ and ω scans	θ_max_ = 28.0°, θ_min_ = 2.2°
Absorption correction: multi-scan (*SADABS*; Bruker, 2009)	*h* = −11→11
	*k* = −22→23
30614 measured reflections	*l* = −17→17
4249 independent reflections	

### (I) Refinement

**Table:**

Refinement on *F*^2^	0 restraints
Least-squares matrix: full	Hydrogen site location: inferred from neighbouring sites
*R*[*F*^2^ > 2σ(*F*^2^)] = 0.054	H-atom parameters constrained
*wR*(*F*^2^) = 0.146	*w* = 1/[σ^2^(*F*_o_^2^) + (0.063*P*)^2^ + 0.2604*P*] where *P* = (*F*_o_^2^ + 2*F*_c_^2^)/3
*S* = 1.08	(Δ/σ)_max_ = 0.001
4249 reflections	Δρ_max_ = 0.17 e Å^−^^3^
236 parameters	Δρ_min_ = −0.19 e Å^−^^3^

### (I) Special details

**Table:**

Geometry. All esds (except the esd in the dihedral angle between two l.s. planes) are estimated using the full covariance matrix. The cell esds are taken into account individually in the estimation of esds in distances, angles and torsion angles; correlations between esds in cell parameters are only used when they are defined by crystal symmetry. An approximate (isotropic) treatment of cell esds is used for estimating esds involving l.s. planes.

### (I) Fractional atomic coordinates and isotropic or equivalent isotropic displacement parameters (Å^2^)

**Table:**

	*x*	*y*	*z*	*U*_iso_*/*U*_eq_	
O1	0.53861 (18)	0.72502 (7)	0.79211 (12)	0.0752 (4)	
O2	0.20209 (16)	1.03186 (6)	0.56513 (10)	0.0642 (3)	
C1	0.53056 (19)	0.86680 (8)	0.94301 (13)	0.0478 (3)	
C2	0.6917 (2)	0.88894 (10)	0.95792 (15)	0.0592 (4)	
H2A	0.7215	0.8740	0.9017	0.071*	
C3	0.8018 (3)	0.93154 (11)	1.05315 (18)	0.0738 (5)	
H3A	0.9052	0.9465	1.0604	0.089*	
C4	0.7618 (3)	0.95337 (12)	1.14117 (18)	0.0802 (6)	
H4A	0.8400	0.9815	1.2071	0.096*	
C5	0.6114 (3)	0.93389 (11)	1.13057 (16)	0.0731 (5)	
H5A	0.5870	0.9487	1.1896	0.088*	
C6	0.4886 (2)	0.89110 (9)	1.03106 (13)	0.0552 (4)	
C7	0.3300 (2)	0.87229 (10)	1.01595 (15)	0.0615 (4)	
H7A	0.3036	0.8876	1.0738	0.074*	
C8	0.2100 (2)	0.83166 (9)	0.91831 (15)	0.0538 (4)	
C9	0.0457 (3)	0.81257 (11)	0.90222 (19)	0.0713 (5)	
H9A	0.0176	0.8282	0.9591	0.086*	
C10	−0.0685 (3)	0.77249 (12)	0.8065 (2)	0.0787 (6)	
H10A	−0.1744	0.7609	0.7977	0.094*	
C11	−0.0284 (2)	0.74811 (11)	0.7196 (2)	0.0757 (6)	
H11A	−0.1087	0.7209	0.6535	0.091*	
C12	0.1255 (2)	0.76379 (9)	0.73108 (16)	0.0605 (4)	
H12A	0.1497	0.7466	0.6730	0.073*	
C13	0.25104 (19)	0.80611 (8)	0.83027 (14)	0.0478 (3)	
C14	0.41240 (18)	0.82319 (8)	0.84557 (13)	0.0445 (3)	
C15	0.4659 (2)	0.78700 (8)	0.76321 (14)	0.0500 (4)	
C16	0.4322 (2)	0.82415 (9)	0.65306 (14)	0.0526 (4)	
H16A	0.4648	0.7987	0.6044	0.063*	
C17	0.35781 (19)	0.89218 (8)	0.61731 (12)	0.0459 (3)	
H17A	0.3278	0.9171	0.6678	0.055*	
C18	0.3182 (2)	0.93191 (9)	0.50726 (13)	0.0489 (4)	
C19	0.3583 (2)	0.90106 (11)	0.42487 (15)	0.0640 (5)	
H19A	0.4128	0.8538	0.4405	0.077*	
C20	0.3189 (3)	0.93900 (14)	0.32089 (17)	0.0804 (6)	
H20A	0.3470	0.9176	0.2670	0.096*	
C21	0.2377 (3)	1.00888 (14)	0.29715 (17)	0.0813 (6)	
H21A	0.2101	1.0343	0.2265	0.098*	
C22	0.1965 (2)	1.04188 (11)	0.37628 (16)	0.0686 (5)	
H22A	0.1424	1.0893	0.3594	0.082*	
C23	0.2366 (2)	1.00373 (9)	0.48149 (13)	0.0527 (4)	
C24	0.1245 (3)	1.10560 (10)	0.54615 (19)	0.0769 (6)	
H24A	0.1028	1.1170	0.6100	0.115*	
H24B	0.0190	1.1057	0.4717	0.115*	
H24C	0.1994	1.1437	0.5440	0.115*	

### (I) Atomic displacement parameters (Å^2^)

**Table:**

	*U*^11^	*U*^22^	*U*^33^	*U*^12^	*U*^13^	*U*^23^
O1	0.0919 (9)	0.0621 (7)	0.0778 (9)	0.0293 (7)	0.0470 (8)	0.0145 (6)
O2	0.0823 (8)	0.0544 (6)	0.0513 (7)	0.0117 (6)	0.0300 (6)	0.0043 (5)
C1	0.0516 (8)	0.0490 (8)	0.0389 (8)	0.0037 (6)	0.0197 (7)	0.0081 (6)
C2	0.0552 (10)	0.0622 (10)	0.0545 (10)	−0.0019 (8)	0.0231 (8)	0.0058 (8)
C3	0.0619 (11)	0.0723 (12)	0.0692 (13)	−0.0113 (9)	0.0191 (10)	0.0034 (10)
C4	0.0840 (15)	0.0739 (12)	0.0522 (12)	−0.0120 (11)	0.0110 (10)	−0.0062 (9)
C5	0.0892 (15)	0.0761 (12)	0.0414 (10)	−0.0018 (10)	0.0231 (10)	−0.0041 (9)
C6	0.0686 (11)	0.0583 (9)	0.0341 (8)	0.0049 (8)	0.0221 (8)	0.0073 (7)
C7	0.0750 (12)	0.0749 (11)	0.0449 (9)	0.0106 (9)	0.0377 (9)	0.0105 (8)
C8	0.0590 (10)	0.0583 (9)	0.0513 (10)	0.0092 (7)	0.0328 (8)	0.0177 (7)
C9	0.0678 (12)	0.0830 (12)	0.0783 (13)	0.0143 (10)	0.0479 (11)	0.0273 (11)
C10	0.0582 (11)	0.0746 (12)	0.1062 (17)	0.0024 (10)	0.0433 (12)	0.0263 (12)
C11	0.0574 (11)	0.0589 (10)	0.0954 (16)	−0.0060 (8)	0.0265 (11)	0.0023 (10)
C12	0.0555 (10)	0.0526 (9)	0.0668 (11)	0.0000 (7)	0.0254 (9)	−0.0020 (8)
C13	0.0499 (8)	0.0445 (7)	0.0482 (9)	0.0056 (6)	0.0241 (7)	0.0103 (6)
C14	0.0485 (8)	0.0440 (7)	0.0398 (8)	0.0056 (6)	0.0211 (7)	0.0077 (6)
C15	0.0499 (8)	0.0483 (8)	0.0502 (9)	0.0035 (7)	0.0237 (7)	0.0006 (7)
C16	0.0621 (10)	0.0557 (9)	0.0459 (9)	0.0033 (7)	0.0315 (8)	−0.0045 (7)
C17	0.0504 (8)	0.0504 (8)	0.0375 (8)	−0.0048 (6)	0.0223 (7)	−0.0066 (6)
C18	0.0541 (9)	0.0543 (8)	0.0354 (8)	−0.0121 (7)	0.0202 (7)	−0.0058 (6)
C19	0.0771 (12)	0.0733 (11)	0.0459 (9)	−0.0143 (9)	0.0340 (9)	−0.0113 (8)
C20	0.0965 (15)	0.1083 (17)	0.0450 (11)	−0.0302 (13)	0.0418 (11)	−0.0149 (11)
C21	0.0902 (15)	0.1026 (16)	0.0408 (10)	−0.0332 (13)	0.0249 (10)	0.0068 (10)
C22	0.0707 (12)	0.0707 (11)	0.0470 (10)	−0.0156 (9)	0.0162 (9)	0.0103 (8)
C23	0.0546 (9)	0.0565 (9)	0.0367 (8)	−0.0135 (7)	0.0149 (7)	−0.0019 (7)
C24	0.0823 (13)	0.0525 (10)	0.0829 (14)	0.0105 (9)	0.0313 (11)	0.0054 (9)

### (I) Geometric parameters (Å, º)

**Table:**

O1—C15	1.2226 (18)	C11—H11A	0.9300
O2—C23	1.3574 (19)	C12—C13	1.423 (2)
O2—C24	1.426 (2)	C12—H12A	0.9300
C1—C14	1.401 (2)	C13—C14	1.405 (2)
C1—C2	1.426 (2)	C14—C15	1.509 (2)
C1—C6	1.431 (2)	C15—C16	1.451 (2)
C2—C3	1.355 (3)	C16—C17	1.327 (2)
C2—H2A	0.9300	C16—H16A	0.9300
C3—C4	1.408 (3)	C17—C18	1.457 (2)
C3—H3A	0.9300	C17—H17A	0.9300
C4—C5	1.343 (3)	C18—C19	1.393 (2)
C4—H4A	0.9300	C18—C23	1.406 (2)
C5—C6	1.421 (3)	C19—C20	1.375 (3)
C5—H5A	0.9300	C19—H19A	0.9300
C6—C7	1.389 (2)	C20—C21	1.376 (3)
C7—C8	1.381 (2)	C20—H20A	0.9300
C7—H7A	0.9300	C21—C22	1.377 (3)
C8—C13	1.433 (2)	C21—H21A	0.9300
C8—C9	1.435 (2)	C22—C23	1.389 (2)
C9—C10	1.346 (3)	C22—H22A	0.9300
C9—H9A	0.9300	C24—H24A	0.9600
C10—C11	1.406 (3)	C24—H24B	0.9600
C10—H10A	0.9300	C24—H24C	0.9600
C11—C12	1.354 (2)		
			
C23—O2—C24	118.55 (13)	C14—C13—C8	119.06 (14)
C14—C1—C2	122.63 (14)	C12—C13—C8	117.99 (14)
C14—C1—C6	119.23 (14)	C1—C14—C13	121.04 (13)
C2—C1—C6	118.13 (15)	C1—C14—C15	119.37 (13)
C3—C2—C1	120.73 (17)	C13—C14—C15	119.27 (13)
C3—C2—H2A	119.6	O1—C15—C16	120.75 (14)
C1—C2—H2A	119.6	O1—C15—C14	117.85 (14)
C2—C3—C4	120.82 (19)	C16—C15—C14	121.40 (13)
C2—C3—H3A	119.6	C17—C16—C15	124.17 (13)
C4—C3—H3A	119.6	C17—C16—H16A	117.9
C5—C4—C3	120.41 (19)	C15—C16—H16A	117.9
C5—C4—H4A	119.8	C16—C17—C18	126.82 (14)
C3—C4—H4A	119.8	C16—C17—H17A	116.6
C4—C5—C6	121.37 (19)	C18—C17—H17A	116.6
C4—C5—H5A	119.3	C19—C18—C23	117.97 (14)
C6—C5—H5A	119.3	C19—C18—C17	122.13 (15)
C7—C6—C5	122.56 (16)	C23—C18—C17	119.90 (13)
C7—C6—C1	118.96 (15)	C20—C19—C18	121.46 (19)
C5—C6—C1	118.48 (16)	C20—C19—H19A	119.3
C8—C7—C6	122.45 (15)	C18—C19—H19A	119.3
C8—C7—H7A	118.8	C19—C20—C21	119.51 (18)
C6—C7—H7A	118.8	C19—C20—H20A	120.2
C7—C8—C13	119.20 (14)	C21—C20—H20A	120.2
C7—C8—C9	122.52 (16)	C20—C21—C22	121.12 (17)
C13—C8—C9	118.27 (17)	C20—C21—H21A	119.4
C10—C9—C8	121.30 (18)	C22—C21—H21A	119.4
C10—C9—H9A	119.4	C21—C22—C23	119.45 (19)
C8—C9—H9A	119.4	C21—C22—H22A	120.3
C9—C10—C11	120.29 (17)	C23—C22—H22A	120.3
C9—C10—H10A	119.9	O2—C23—C22	123.70 (16)
C11—C10—H10A	119.9	O2—C23—C18	115.80 (13)
C12—C11—C10	120.88 (19)	C22—C23—C18	120.49 (16)
C12—C11—H11A	119.6	O2—C24—H24A	109.5
C10—C11—H11A	119.6	O2—C24—H24B	109.5
C11—C12—C13	121.27 (18)	H24A—C24—H24B	109.5
C11—C12—H12A	119.4	O2—C24—H24C	109.5
C13—C12—H12A	119.4	H24A—C24—H24C	109.5
C14—C13—C12	122.95 (14)	H24B—C24—H24C	109.5
			
C14—C1—C2—C3	179.60 (15)	C2—C1—C14—C15	10.2 (2)
C6—C1—C2—C3	0.2 (2)	C6—C1—C14—C15	−170.46 (13)
C1—C2—C3—C4	1.6 (3)	C12—C13—C14—C1	178.29 (13)
C2—C3—C4—C5	−1.7 (3)	C8—C13—C14—C1	−2.2 (2)
C3—C4—C5—C6	−0.1 (3)	C12—C13—C14—C15	−8.3 (2)
C4—C5—C6—C7	−177.89 (18)	C8—C13—C14—C15	171.27 (13)
C4—C5—C6—C1	1.9 (3)	C1—C14—C15—O1	84.19 (18)
C14—C1—C6—C7	−1.5 (2)	C13—C14—C15—O1	−89.38 (19)
C2—C1—C6—C7	177.85 (14)	C1—C14—C15—C16	−95.91 (18)
C14—C1—C6—C5	178.68 (14)	C13—C14—C15—C16	90.53 (18)
C2—C1—C6—C5	−1.9 (2)	O1—C15—C16—C17	−178.25 (16)
C5—C6—C7—C8	179.05 (16)	C14—C15—C16—C17	1.8 (2)
C1—C6—C7—C8	−0.7 (2)	C15—C16—C17—C18	−178.99 (14)
C6—C7—C8—C13	1.5 (2)	C16—C17—C18—C19	−0.7 (2)
C6—C7—C8—C9	−179.64 (16)	C16—C17—C18—C23	179.15 (15)
C7—C8—C9—C10	−179.61 (17)	C23—C18—C19—C20	−0.3 (3)
C13—C8—C9—C10	−0.8 (3)	C17—C18—C19—C20	179.50 (16)
C8—C9—C10—C11	0.2 (3)	C18—C19—C20—C21	−0.2 (3)
C9—C10—C11—C12	0.6 (3)	C19—C20—C21—C22	0.6 (3)
C10—C11—C12—C13	−0.8 (3)	C20—C21—C22—C23	−0.5 (3)
C11—C12—C13—C14	179.68 (15)	C24—O2—C23—C22	−1.7 (2)
C11—C12—C13—C8	0.1 (2)	C24—O2—C23—C18	177.97 (15)
C7—C8—C13—C14	−0.1 (2)	C21—C22—C23—O2	179.57 (16)
C9—C8—C13—C14	−178.96 (13)	C21—C22—C23—C18	−0.1 (2)
C7—C8—C13—C12	179.47 (14)	C19—C18—C23—O2	−179.20 (14)
C9—C8—C13—C12	0.6 (2)	C17—C18—C23—O2	0.9 (2)
C2—C1—C14—C13	−176.37 (13)	C19—C18—C23—C22	0.5 (2)
C6—C1—C14—C13	3.0 (2)	C17—C18—C23—C22	−179.34 (14)

### (E)-1-(Anthracen-9-yl)-3-(3-fluoro-4-methoxyphenyl)prop-2-en-1-one (II) Crystal data

**Table:**

C_24_H_17_FO_2_	*Z* = 2
*M**_r_* = 356.37	*F*(000) = 372
Triclinic, *P*1	*D*_x_ = 1.308 Mg m^−^^3^
*a* = 8.6646 (5) Å	Mo *K*α radiation, λ = 0.71073 Å
*b* = 9.5752 (5) Å	Cell parameters from 8625 reflections
*c* = 11.5636 (6) Å	θ = 2.2–30.2°
α = 100.593 (2)°	µ = 0.09 mm^−^^1^
β = 105.443 (2)°	*T* = 296 K
γ = 92.422 (2)°	Block, yellow
*V* = 904.76 (9) Å^3^	0.99 × 0.31 × 0.25 mm

### (E)-1-(Anthracen-9-yl)-3-(3-fluoro-4-methoxyphenyl)prop-2-en-1-one (II) Data collection

**Table:**

Bruker SMART APEXII DUO CCD area-detector diffractometer	3307 reflections with *I* > 2σ(*I*)
Radiation source: fine-focus sealed tube	*R*_int_ = 0.045
φ and ω scans	θ_max_ = 30.4°, θ_min_ = 1.9°
Absorption correction: multi-scan (*SADABS*; Bruker, 2009)	*h* = −12→12
	*k* = −13→13
34988 measured reflections	*l* = −16→16
5394 independent reflections	

### (E)-1-(Anthracen-9-yl)-3-(3-fluoro-4-methoxyphenyl)prop-2-en-1-one (II) Refinement

**Table:**

Refinement on *F*^2^	0 restraints
Least-squares matrix: full	Hydrogen site location: inferred from neighbouring sites
*R*[*F*^2^ > 2σ(*F*^2^)] = 0.059	H-atom parameters constrained
*wR*(*F*^2^) = 0.183	*w* = 1/[σ^2^(*F*_o_^2^) + (0.0672*P*)^2^ + 0.4075*P*] where *P* = (*F*_o_^2^ + 2*F*_c_^2^)/3
*S* = 1.02	(Δ/σ)_max_ < 0.001
5394 reflections	Δρ_max_ = 0.27 e Å^−^^3^
245 parameters	Δρ_min_ = −0.20 e Å^−^^3^

### (E)-1-(Anthracen-9-yl)-3-(3-fluoro-4-methoxyphenyl)prop-2-en-1-one (II) Special details

**Table:**

Geometry. All esds (except the esd in the dihedral angle between two l.s. planes) are estimated using the full covariance matrix. The cell esds are taken into account individually in the estimation of esds in distances, angles and torsion angles; correlations between esds in cell parameters are only used when they are defined by crystal symmetry. An approximate (isotropic) treatment of cell esds is used for estimating esds involving l.s. planes.

### (E)-1-(Anthracen-9-yl)-3-(3-fluoro-4-methoxyphenyl)prop-2-en-1-one (II) Fractional atomic coordinates and isotropic or equivalent isotropic displacement parameters (Å^2^)

**Table:**

	*x*	*y*	*z*	*U*_iso_*/*U*_eq_	
F1	−0.05579 (15)	0.41714 (15)	0.24083 (12)	0.0805 (4)	
O1	0.1312 (2)	−0.03230 (17)	0.68989 (16)	0.0842 (6)	
O2	0.12761 (17)	0.65677 (16)	0.28147 (14)	0.0642 (4)	
C1	0.49638 (19)	0.04101 (17)	0.81471 (15)	0.0403 (4)	
C2	0.5049 (3)	−0.0702 (2)	0.71696 (18)	0.0572 (5)	
H2A	0.4185	−0.0931	0.6467	0.069*	
C3	0.6364 (3)	−0.1434 (2)	0.7244 (2)	0.0700 (6)	
H3A	0.6391	−0.2152	0.6589	0.084*	
C4	0.7689 (3)	−0.1126 (2)	0.8295 (2)	0.0661 (6)	
H4A	0.8579	−0.1644	0.8333	0.079*	
C5	0.7672 (2)	−0.0079 (2)	0.92466 (19)	0.0548 (5)	
H5A	0.8551	0.0111	0.9940	0.066*	
C6	0.63319 (19)	0.07396 (18)	0.92079 (15)	0.0409 (4)	
C7	0.63115 (19)	0.18423 (19)	1.01674 (15)	0.0436 (4)	
H7A	0.7199	0.2054	1.0854	0.052*	
C8	0.5005 (2)	0.26367 (18)	1.01297 (14)	0.0413 (4)	
C9	0.5007 (2)	0.3788 (2)	1.11086 (17)	0.0556 (5)	
H9A	0.5913	0.4024	1.1779	0.067*	
C10	0.3724 (3)	0.4537 (2)	1.1079 (2)	0.0659 (6)	
H10A	0.3746	0.5280	1.1728	0.079*	
C11	0.2345 (3)	0.4199 (2)	1.0066 (2)	0.0655 (6)	
H11A	0.1461	0.4719	1.0059	0.079*	
C12	0.2286 (2)	0.3125 (2)	0.90999 (18)	0.0533 (4)	
H12A	0.1364	0.2924	0.8440	0.064*	
C13	0.36166 (19)	0.23022 (17)	0.90848 (15)	0.0399 (3)	
C14	0.36231 (19)	0.11926 (17)	0.81074 (14)	0.0390 (3)	
C15	0.2162 (2)	0.07711 (19)	0.70129 (17)	0.0494 (4)	
C16	0.1775 (2)	0.1640 (2)	0.60830 (16)	0.0516 (4)	
H16A	0.0893	0.1315	0.5407	0.062*	
C17	0.2592 (2)	0.28637 (19)	0.61334 (15)	0.0442 (4)	
H17A	0.3492	0.3150	0.6803	0.053*	
C18	0.22475 (19)	0.38103 (18)	0.52646 (14)	0.0412 (4)	
C19	0.0943 (2)	0.35137 (19)	0.42003 (16)	0.0453 (4)	
H19A	0.0259	0.2681	0.4022	0.054*	
C20	0.0689 (2)	0.4455 (2)	0.34319 (16)	0.0477 (4)	
C21	0.1660 (2)	0.57237 (19)	0.36488 (16)	0.0453 (4)	
C22	0.2941 (2)	0.6011 (2)	0.46933 (17)	0.0498 (4)	
H22A	0.3621	0.6847	0.4869	0.060*	
C23	0.3221 (2)	0.50661 (19)	0.54785 (15)	0.0481 (4)	
H23A	0.4094	0.5281	0.6175	0.058*	
C24	0.2160 (3)	0.7934 (3)	0.3095 (3)	0.0818 (7)	
H24D	0.1755	0.8436	0.2447	0.123*	
H24A	0.3276	0.7817	0.3177	0.123*	
H24B	0.2043	0.8470	0.3850	0.123*	

### (E)-1-(Anthracen-9-yl)-3-(3-fluoro-4-methoxyphenyl)prop-2-en-1-one (II) Atomic displacement parameters (Å^2^)

**Table:**

	*U*^11^	*U*^22^	*U*^33^	*U*^12^	*U*^13^	*U*^23^
F1	0.0667 (8)	0.0865 (9)	0.0653 (8)	−0.0140 (7)	−0.0250 (6)	0.0267 (7)
O1	0.0745 (10)	0.0659 (10)	0.0862 (11)	−0.0277 (8)	−0.0240 (8)	0.0265 (8)
O2	0.0579 (8)	0.0682 (9)	0.0672 (9)	0.0042 (7)	0.0063 (7)	0.0323 (7)
C1	0.0414 (8)	0.0376 (8)	0.0392 (8)	−0.0023 (6)	0.0078 (6)	0.0073 (6)
C2	0.0593 (11)	0.0508 (10)	0.0517 (10)	0.0007 (9)	0.0088 (9)	−0.0033 (8)
C3	0.0745 (15)	0.0583 (12)	0.0732 (14)	0.0090 (11)	0.0272 (12)	−0.0069 (11)
C4	0.0548 (12)	0.0603 (12)	0.0862 (16)	0.0168 (10)	0.0260 (11)	0.0099 (11)
C5	0.0412 (9)	0.0592 (11)	0.0631 (12)	0.0090 (8)	0.0107 (8)	0.0148 (9)
C6	0.0360 (8)	0.0430 (8)	0.0430 (8)	0.0015 (6)	0.0078 (6)	0.0118 (7)
C7	0.0357 (8)	0.0511 (9)	0.0380 (8)	0.0005 (7)	0.0010 (6)	0.0086 (7)
C8	0.0413 (8)	0.0440 (9)	0.0353 (8)	0.0010 (7)	0.0054 (6)	0.0080 (6)
C9	0.0580 (11)	0.0593 (11)	0.0410 (9)	0.0038 (9)	0.0063 (8)	0.0002 (8)
C10	0.0721 (14)	0.0636 (13)	0.0555 (12)	0.0125 (11)	0.0162 (10)	−0.0036 (10)
C11	0.0611 (12)	0.0653 (13)	0.0708 (14)	0.0223 (10)	0.0195 (10)	0.0102 (11)
C12	0.0448 (9)	0.0571 (11)	0.0535 (10)	0.0096 (8)	0.0043 (8)	0.0127 (8)
C13	0.0373 (8)	0.0400 (8)	0.0399 (8)	0.0028 (6)	0.0050 (6)	0.0104 (6)
C14	0.0379 (8)	0.0378 (8)	0.0367 (8)	−0.0014 (6)	0.0015 (6)	0.0102 (6)
C15	0.0455 (9)	0.0454 (9)	0.0474 (9)	−0.0023 (7)	−0.0021 (7)	0.0083 (7)
C16	0.0462 (9)	0.0560 (11)	0.0412 (9)	−0.0004 (8)	−0.0056 (7)	0.0085 (8)
C17	0.0378 (8)	0.0524 (10)	0.0355 (8)	0.0037 (7)	0.0019 (6)	0.0039 (7)
C18	0.0384 (8)	0.0469 (9)	0.0349 (8)	0.0055 (7)	0.0076 (6)	0.0036 (7)
C19	0.0417 (8)	0.0443 (9)	0.0440 (9)	−0.0001 (7)	0.0048 (7)	0.0057 (7)
C20	0.0379 (8)	0.0561 (10)	0.0411 (9)	0.0029 (7)	−0.0011 (7)	0.0077 (7)
C21	0.0419 (9)	0.0502 (10)	0.0451 (9)	0.0073 (7)	0.0115 (7)	0.0128 (7)
C22	0.0485 (10)	0.0479 (10)	0.0490 (10)	−0.0036 (8)	0.0097 (8)	0.0072 (8)
C23	0.0432 (9)	0.0553 (10)	0.0372 (8)	−0.0027 (8)	0.0017 (7)	0.0037 (7)
C24	0.0764 (16)	0.0712 (15)	0.104 (2)	−0.0004 (12)	0.0183 (14)	0.0456 (14)

### (E)-1-(Anthracen-9-yl)-3-(3-fluoro-4-methoxyphenyl)prop-2-en-1-one (II) Geometric parameters (Å, º)

**Table:**

F1—C20	1.3485 (19)	C11—C12	1.361 (3)
O1—C15	1.220 (2)	C11—H11A	0.9300
O2—C21	1.354 (2)	C12—C13	1.426 (2)
O2—C24	1.425 (3)	C12—H12A	0.9300
C1—C14	1.404 (2)	C13—C14	1.403 (2)
C1—C2	1.424 (2)	C14—C15	1.511 (2)
C1—C6	1.436 (2)	C15—C16	1.457 (3)
C2—C3	1.354 (3)	C16—C17	1.327 (3)
C2—H2A	0.9300	C16—H16A	0.9300
C3—C4	1.407 (3)	C17—C18	1.455 (2)
C3—H3A	0.9300	C17—H17A	0.9300
C4—C5	1.349 (3)	C18—C23	1.383 (2)
C4—H4A	0.9300	C18—C19	1.406 (2)
C5—C6	1.424 (2)	C19—C20	1.363 (3)
C5—H5A	0.9300	C19—H19A	0.9300
C6—C7	1.388 (2)	C20—C21	1.391 (3)
C7—C8	1.386 (2)	C21—C22	1.380 (2)
C7—H7A	0.9300	C22—C23	1.380 (3)
C8—C9	1.427 (2)	C22—H22A	0.9300
C8—C13	1.436 (2)	C23—H23A	0.9300
C9—C10	1.345 (3)	C24—H24D	0.9600
C9—H9A	0.9300	C24—H24A	0.9600
C10—C11	1.409 (3)	C24—H24B	0.9600
C10—H10A	0.9300		
			
C21—O2—C24	117.53 (17)	C14—C13—C8	119.19 (14)
C14—C1—C2	123.01 (15)	C12—C13—C8	117.62 (15)
C14—C1—C6	119.47 (15)	C13—C14—C1	120.71 (14)
C2—C1—C6	117.52 (16)	C13—C14—C15	120.79 (15)
C3—C2—C1	121.23 (19)	C1—C14—C15	118.45 (15)
C3—C2—H2A	119.4	O1—C15—C16	119.73 (16)
C1—C2—H2A	119.4	O1—C15—C14	119.46 (16)
C2—C3—C4	121.1 (2)	C16—C15—C14	120.80 (15)
C2—C3—H3A	119.5	C17—C16—C15	124.58 (16)
C4—C3—H3A	119.5	C17—C16—H16A	117.7
C5—C4—C3	120.13 (19)	C15—C16—H16A	117.7
C5—C4—H4A	119.9	C16—C17—C18	127.67 (15)
C3—C4—H4A	119.9	C16—C17—H17A	116.2
C4—C5—C6	121.11 (19)	C18—C17—H17A	116.2
C4—C5—H5A	119.4	C23—C18—C19	117.44 (16)
C6—C5—H5A	119.4	C23—C18—C17	119.75 (14)
C7—C6—C5	121.83 (16)	C19—C18—C17	122.81 (15)
C7—C6—C1	119.24 (15)	C20—C19—C18	119.55 (16)
C5—C6—C1	118.93 (16)	C20—C19—H19A	120.2
C8—C7—C6	121.79 (15)	C18—C19—H19A	120.2
C8—C7—H7A	119.1	F1—C20—C19	119.69 (16)
C6—C7—H7A	119.1	F1—C20—C21	117.16 (16)
C7—C8—C9	121.43 (15)	C19—C20—C21	123.15 (15)
C7—C8—C13	119.59 (15)	O2—C21—C22	125.48 (17)
C9—C8—C13	118.98 (16)	O2—C21—C20	117.35 (15)
C10—C9—C8	121.19 (18)	C22—C21—C20	117.17 (16)
C10—C9—H9A	119.4	C23—C22—C21	120.44 (17)
C8—C9—H9A	119.4	C23—C22—H22A	119.8
C9—C10—C11	120.17 (19)	C21—C22—H22A	119.8
C9—C10—H10A	119.9	C22—C23—C18	122.25 (15)
C11—C10—H10A	119.9	C22—C23—H23A	118.9
C12—C11—C10	121.12 (19)	C18—C23—H23A	118.9
C12—C11—H11A	119.4	O2—C24—H24D	109.5
C10—C11—H11A	119.4	O2—C24—H24A	109.5
C11—C12—C13	120.89 (17)	H24D—C24—H24A	109.5
C11—C12—H12A	119.6	O2—C24—H24B	109.5
C13—C12—H12A	119.6	H24D—C24—H24B	109.5
C14—C13—C12	123.18 (15)	H24A—C24—H24B	109.5
			
C14—C1—C2—C3	−179.92 (19)	C8—C13—C14—C15	177.67 (15)
C6—C1—C2—C3	0.8 (3)	C2—C1—C14—C13	−178.46 (16)
C1—C2—C3—C4	0.4 (4)	C6—C1—C14—C13	0.8 (2)
C2—C3—C4—C5	−0.6 (4)	C2—C1—C14—C15	4.1 (2)
C3—C4—C5—C6	−0.5 (3)	C6—C1—C14—C15	−176.59 (15)
C4—C5—C6—C7	−178.44 (19)	C13—C14—C15—O1	−104.9 (2)
C4—C5—C6—C1	1.7 (3)	C1—C14—C15—O1	72.5 (3)
C14—C1—C6—C7	−1.0 (2)	C13—C14—C15—C16	76.3 (2)
C2—C1—C6—C7	178.36 (16)	C1—C14—C15—C16	−106.3 (2)
C14—C1—C6—C5	178.85 (16)	O1—C15—C16—C17	178.2 (2)
C2—C1—C6—C5	−1.8 (2)	C14—C15—C16—C17	−3.0 (3)
C5—C6—C7—C8	−179.87 (16)	C15—C16—C17—C18	−177.76 (17)
C1—C6—C7—C8	0.0 (3)	C16—C17—C18—C23	178.73 (18)
C6—C7—C8—C9	−178.66 (17)	C16—C17—C18—C19	−0.9 (3)
C6—C7—C8—C13	1.2 (3)	C23—C18—C19—C20	0.0 (3)
C7—C8—C9—C10	−178.64 (19)	C17—C18—C19—C20	179.61 (16)
C13—C8—C9—C10	1.5 (3)	C18—C19—C20—F1	179.80 (16)
C8—C9—C10—C11	−0.4 (3)	C18—C19—C20—C21	−0.5 (3)
C9—C10—C11—C12	−0.5 (4)	C24—O2—C21—C22	−6.9 (3)
C10—C11—C12—C13	0.3 (3)	C24—O2—C21—C20	173.58 (19)
C11—C12—C13—C14	−179.42 (18)	F1—C20—C21—O2	−0.1 (3)
C11—C12—C13—C8	0.8 (3)	C19—C20—C21—O2	−179.88 (17)
C7—C8—C13—C14	−1.3 (2)	F1—C20—C21—C22	−179.69 (17)
C9—C8—C13—C14	178.55 (16)	C19—C20—C21—C22	0.6 (3)
C7—C8—C13—C12	178.46 (16)	O2—C21—C22—C23	−179.74 (17)
C9—C8—C13—C12	−1.7 (2)	C20—C21—C22—C23	−0.2 (3)
C12—C13—C14—C1	−179.46 (16)	C21—C22—C23—C18	−0.2 (3)
C8—C13—C14—C1	0.3 (2)	C19—C18—C23—C22	0.3 (3)
C12—C13—C14—C15	−2.1 (3)	C17—C18—C23—C22	−179.30 (16)

### (E)-1-(Anthracen-9-yl)-3-(3-fluoro-4-methoxyphenyl)prop-2-en-1-one (II) Hydrogen-bond geometry (Å, º)

Cg4 is the centroid of the C1–C6 ring.

**Table:**

*D*—H···*A*	*D*—H	H···*A*	*D*···*A*	*D*—H···*A*
C12—H12*A*···O2^i^	0.93	2.48	3.345 (2)	154
C19—H19*A*···O1^ii^	0.93	2.48	3.393 (3)	166
C24—H24*D*···*Cg*4^iii^	0.96	2.77	3.391 (3)	123

Symmetry codes: (i) −*x*, −*y*+1, −*z*+1; (ii) −*x*, −*y*, −*z*+1; (iii) −*x*+1, −*y*+1, −*z*+1.
