# Supplementary material for: Mol­ecular structure, DFT studies and UV–Vis absorption of two new linear fused ring chalcones: (E)-1-(anthracen-9-yl)-3-(2-meth­oxy­phen­yl)prop-2-en-1-one and (E)-1-(anthracen-9-yl)-3-(3-fluoro-4-meth­oxy­phen­yl)prop-2-en-1-one
Source: Acta Crystallogr E Crystallogr Commun. 2018 Jul 13;74(Pt 8):1087–92. doi: 10.1107/S205698901800974X (PMC6072983; doi:10.1107/S205698901800974X)
Supplement: Supplementary file 6 [file e-74-01087-sup6.pdf]

Molecular structure, DFT studies and UV-Vis absorption of two new linear fused ring chalcone: *(E)*-1-(anthracen-9-yl)-3-(2-methoxyphenyl)prop-2-en-1-one and *(E)*-1-(anthracen-9-yl)-3-(3-fluoro-4-methoxyphenyl)prop-2-en-1-one

Supplementary Materials

Table S1 A Comparison Selected Single Crystal X-Ray data and DFT Geometry Optimized Data for Compounds (I) and (II).

	**Compound I**		**Compound II**	
**Bonds**	**X-ray (Experimental) ($Å^0$)**	**DFT* (Theoretical) ($Å^0$)**	**X-ray (Experimental) ($Å^0$)**	**DFT* (Theoretical) ($Å^0$)**
C15-O1	1.22 (18)	1.22	1.22 (2)	1.22
C1-C14	1.40 (2)	1.40	1.40 (2)	1.41
C1-C2	1.43 (2)	1.43	1.42 (2)	1.43
C2-C3	1.36 (3)	1.35	1.35 (3)	1.37
C3-C4	1.41 (3)	1.41	1.41 (3)	1.42
C4-C5	1.34 (3)	1.34	1.35 (3)	1.37
C5-C6	1.42 (3)	1.42	1.42 (2)	1.43
C6-C7	1.39 (2)	1.39	1.39 (2)	1.40
C7-C8	1.38 (2)	1.38	1.39 (2)	1.40
C8-C9	1.44 (2)	1.43	1.43 (2)	1.43
C9-C10	1.35 (3)	1.35	1.35 (3)	1.37
C10-C11	1.41 (3)	1.41	1.41 (3)	1.42
C11-C12	1.35 (2)	1.35	1.36 (3)	1.37
C12-C13	1.42 (2)	1.42	1.43 (2)	1.43
C13-C14	1.41 (2)	1.41	1.40 (2)	1.41
C14-C15	1.51 (2)	1.51	1.51 (2)	1.52
C15-C16	1.45 (2)	1.45	1.46 (3)	1.47
C16-C17	1.33 (2)	1.33	1.33 (3)	1.35
C17-C18	1.46 (2)	1.46	1.46 (2)	1.46
C14—C15—C16	121.40 (13)	121.38	120.80 (15)	119.42
O1—C15—C14	117.85 (14)	117.84	119.46 (16)	119.97
O1—C15—C16	120.75 (14)	120.78	119.73 (16)	120.61
C15—C16—C17	124.17 (13)	124.18	124.58 (16)	124.08
C16—C17—C18	126.82 (14)	126.83	127.67 (15)	127.55

C1—C14—C15—O1	84.19 (18)	84.21	72.5 (3)	88.74
C13—C14—C15—O1	−89.38 (19)	-89.35	−104.9 (2)	-88.72
C1—C14—C15—C16	−95.91 (18)	-95.94	−106.3 (2)	-91.27
C13—C14—C15—C16	90.53 (18)	90.50	76.3 (2)	91.27
O1—C15—C16—C17	−178.25 (16)	-178.26	178.2 (2)	180.00
C14—C15—C16—C17	1.8 (2)	1.89	−3.0 (3)	-2.00
C15—C16—C17—C18	−178.99 (14)	-179.00	−177.76 (17)	179.99
C16—C17—C18—C19	−0.7 (2)	-0.72	−0.9 (3)	0.01
C16—C17—C18—C23	179.15 (15)	179.17	178.73 (18)	-179.99

* B3LPY/6-311++G(d,p)
